# Viromers as carriers for mRNA-mediated expression of therapeutic molecules under inflammatory conditions

**DOI:** 10.1038/s41598-020-72004-8

**Published:** 2020-09-15

**Authors:** Edith Jansig, Stefanie Geissler, Vera Rieckmann, Anja Kuenemund, Benjamin Hietel, Mathias Schenk, Sebastian Wussow, Patrick Kreideweiss, Steffen Panzner, Christian Reinsch, Holger Cynis

**Affiliations:** 1 Department of Drug Design and Target Validation, Fraunhofer Institute for Cell Therapy and Immunology, Weinbergweg 22, 06120 Halle, Germany; 2 BioNTech Delivery Technologies GmbH, Weinbergweg 23, 06120 Halle, Germany

**Keywords:** Applied immunology, Biologics, Gene delivery, Gene therapy, Nucleic-acid therapeutics, Chemokines, Preclinical research, Molecular medicine

## Abstract

Therapeutic mRNA delivery has been described for several treatment options, such as vaccination and cancer immunotherapy. However, mRNA delivery has to be accompanied by the development and testing of suitable carrier materials due to the instability of mRNAs in human body fluids. In the present study, we investigated the ability of recently developed Viromers to deliver mRNAs in a classical inflammatory setting. We tested mRNAs coding for active components of preclinical (7ND) and approved (sTNF-RII) biologics, in vitro and in vivo. 7ND is an established blocker of the CCR2 axis, whereas sTNF-RII is the active component of the approved drug Etanercept. Viromer/mRNA complexes were transfected into murine macrophages in vitro. Protein expression was analysed using Luciferase reporter expression and mainly identified in spleen, blood and bone marrow in vivo. 7ND-mRNA delivery led to efficient blockage of monocytes infiltration in thioglycolate-induced peritonitis in mice, underlining the ability of Viromers to deliver a therapeutic mRNA cargo without overt toxicity. Therefore, we propose Viromer-based mRNA delivery as a suitable option for the treatment of inflammatory disorders beyond infusion of biological molecules.

## Introduction

Cytokines play a pivotal role in inflammatory reactions as mediators of cellular functions. To treat inflammatory disorders, a number of biotechnologically manufactured and thus complex and expensive molecules are approved, e.g. to reduce inflammatory processes in rheumatoid arthritis^1^ or multiple sclerosis^2^. Alternative and presumably more cost-efficient thearapeutic strategies could include expression of biological drugs directly in the patient by means of transient gene therapy^3^.

Transient gene therapy includes the replacement of defective genes by the introduction of intact copies, or the inactivation of pathogenic gene products without a permanent change of the genetic code in somatic cells^4^. Synthetic mRNAs are under investigation as a novel class of transient gene therapeutics^5^. Due to its non-replicative nature and the required delivery to the cytoplasm, synthetic mRNAs have minimal risk of genomic insertional mutagenesis. Recent chemical modifications of mRNAs provide enhanced stability and increased expression levels in the cell as well as reduced inflammatory response^5^. However, the key obstacle in the development of mRNA-based gene therapies is the transport of the genetic material into the target cells in vivo. Synthetic mRNA is readily degraded in most biological fluids and a non-assisted delivery into cells is currently limited to local injections and directed mainly to dendritic cells^6^.

Mostly, lipid nanoparticles (LNPs) are used for delivery of mRNAs into somatic cells. These cationic carriers, although pegylated, interact with serum components or the complement system, and therefore, may induce undesired immune responses^7^.

Viromers, a recently described class of polymers consist of a polycationic core of polyethyleneimine, which is tightly substituted with hydrophobic and anionic side chains. The cationic core enables the binding of nucleic acids and due to an almost neutral surface charge, Viromers are, in contrast to LNPs, compatible with serum and do not form undesirable aggregates. Following uptake of Viromer/nucleic acid complexes and subsequent enrichment in endosomes around the nucleus, acidification of the complex, leads to enhanced hydrophobicity at the particle surface, which in turn mediates endosomal discharge of the complexes and release of nucleic acids into the cytoplasm. This mechanism was described for the surface protein hemagglutinin of the influenza virus.

Viromers have been tested in a number of cell types, including primary and suspension cells^8,9^. The best transfection success has been observed in monocytes and macrophages, since these cells possess efficient endocytic machinery, due to their physiological function^10,11^. Based on their physico-chemical properties, Viromers should deliver cargo molecules more efficiently in vivo compared to other molecules.

Therefore, the aim of the present study was to investigate the suitability of Viromers as carrier material for transient gene therapy in vivo. For this, mRNAs encoding an antagonist of CCR2 (7ND) and encoding soluble TNF receptor II (sTNF-RII) were analysed. In this regard, 7ND is a genetically modified CCL2 molecule lacking amino acids 2–8^12^. Despite the altered N-terminus, 7ND is able to form a heterodimer with native CCL2 molecules, but binds as monomer to CCR2^13^. Since CCR2 is activated only by N-terminally intact CCL2^14^, the receptor is blocked by the antagonistic binding of 7ND. Without the activation of CCR2, monocyte chemotaxis is suppressed and inflammatory response in chronic diseases is reduced^15–18^. In addition, sTNF-RII is a component of the approved biological drug Etanercept, responsible for neutralization of TNF-alpha, a key mediator in inflammatory reactions^1^. Due to the broad understanding of CCL2 and sTNF-RII biology, these molecules were used as prototypic mRNAs to be delivered by Viromers, and the resulting complexes were evaluated for efficacy and safety in the inflammatory animal model of thioglycolate-induced peritonitis^18,19^.

## Results

### Expression of target mRNAs in RAW264.7 cells in vitro

**Figure 1:**
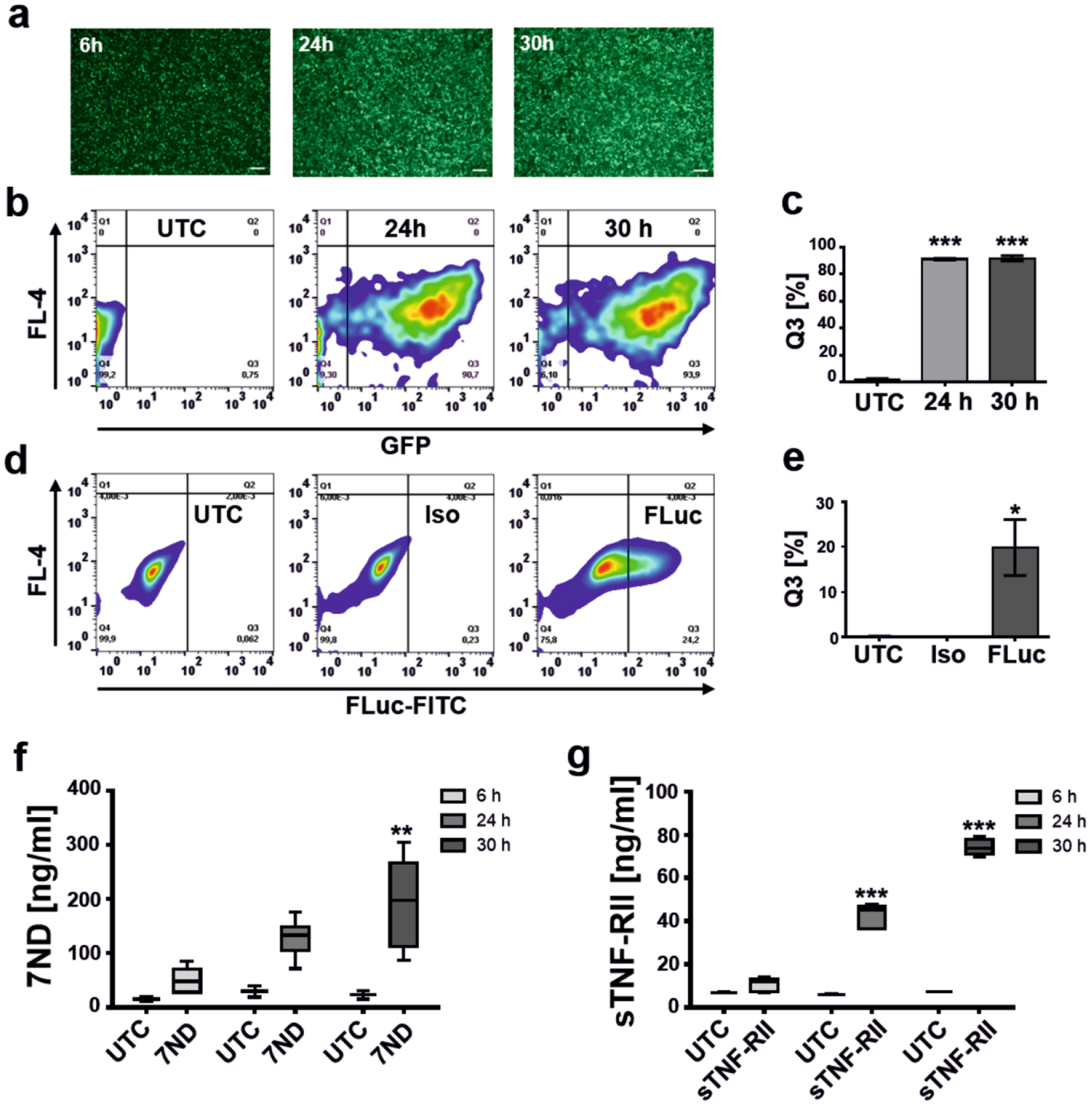
Viromers efficiently transfect murine macrophages. (**a**) Time-dependent appearance of GFP fluorescence in RAW264.7 macrophages observed by fluorescence microscopy after 6 h, 24 h and 30 h post transfection. Scale bar: 100 µm. (**b**,**c**) FACS analysis 24 h and 30 h after transfection of RAW264.7 macrophages using GFP-mRNA (100 ng/well) in comparison to untreated cells (UTC) and quantification (**c**) of GFP-positive cells in Q3 of the FACS plot. Mean ± SD, one-way ANOVA, Dunnett test, ***p < 0.001, n = 3. (**d**,**e**) Intracellular FLuc reporter gene expression (100 ng/well) analysed by FACS 24 h after transfection in comparison to UTC and isotype control (Iso) and quantification of FLuc-positive cells in Q3 of the FACS plot (**e**). Mean ± SD, one-way ANOVA, Dunnett test, *p < 0.05, n = 2. (**f**) Expression of 7ND (100 ng/well) in RAW264.7 macrophages analysed by ELISA 6 h, 24 h and 30 h post transfection. Graph shows box and whiskers (min to max), line at mean, two-way ANOVA, Sidak multiple comparison test, **p < 0.01, n = 2 (UTC), n = 6 (7ND). (**g**) Expression of sTNF-RII (100 ng/well) in RAW264.7 macrophages analysed by ELISA 6 h, 24 h and 30 h post transfection. Graph shows box and whiskers (min to max), line at mean, two-way ANOVA, Sidak multiple comparison test, ***p < 0.001, n = 2 (UTC), n = 6 (7ND).

The efficiency of Viromer-mediated mRNA-delivery was first analysed in cell culture using the mouse monocyte/macrophage cell line RAW264.7. The physico-chemical properties of the Viromer/CCL2 and Viromer/FLuc complexes were demonstrated to be optimal (Supplementary Table 1, Supplementary Fig. 1). The cells were transfected using a Viromer/GFP-mRNA-complex and a Viromer/FLuc-mRNA-complex. Afterwards, the GFP- and FLuc-expressing cells were analysed by fluorescence microscopy and by FACS. As determined by fluorescence microscopy (Fig. 1a) and FACS analysis (Fig. 1b), the cells showed high GFP expression (Fig. 1b, c) compared with controls. The increase in FLuc expression was mRNA-dose dependent (Supplementary Fig. 2a, b) and limited by the applied amount of mRNA to 20% positive cells (Fig. 1d, e). Therefore, the applied Viromer-RED was found to efficiently deliver mRNAs to RAW264.7 cells.

**Figure 2:**
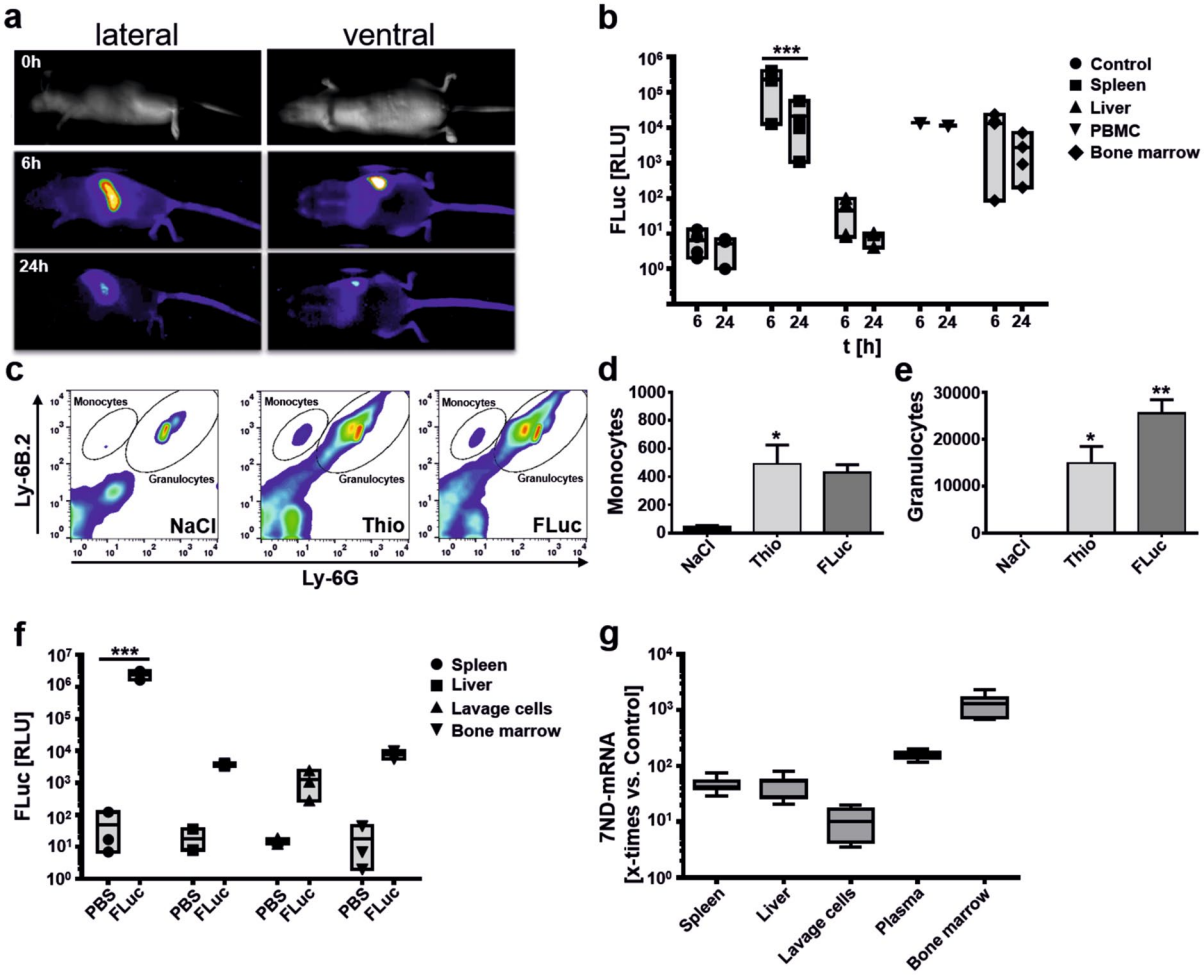
Spleen and bone marrow are major target sites for Viromer delivery. (**a**) Biodistribution of Viromer-mediated FLuc-mRNA delivery analysed by in vivo bioluminescence imaging. Images were taken 6 h and 24 h after i.v. injection of complexes. The lateral view highlights a substantial uptake of Viromer/FLuc by spleen cells, which is supported by the ventral view (no uptake into liver or lungs visible). Minor expression is visible in the upper backbone, tail root, vertebrae and bones. The expression of luciferase is transient. The maximum of FLuc signal was detected after 6 h and signals decrease afterwards. (**b**) Quantification of FLuc expression in different tissues after i.v. application of Viromer/FLuc-mRNA-complexes. Graphs displayed as floating bars (min to max). Line represents the mean. ***p < 0.001, two-way ANOVA, Sidak multiple comparison test. n = 1 (PBMC), n = 4 (bone marrow, control, spleen, liver). (**c**) FACS analysis of cells present in lavage after thioglycolate-induced peritonitis. Representative FACS plots of the groups injected with NaCl, Thioglycolate (Thio) and Thioglycolate and FLuc-mRNA (FLuc) are depicted. Markers Ly-6B.2 (granulocytes, monocytes) and Ly-6G (granulocytes) were used to separate the two populations. (**d**,**e**) Quantification of infiltrating monocytes (**d**) and granulocytes (**e**) using TruCount tubes based on counting 10.000 beads/tube. Mean ± SEM, one-way ANOVA, Dunnett test, *p < 0.05, **p < 0.01, n = 2–3. (**f**) Biodistribution of FLuc in the model of thioglycolate-induced peritonitis after i.v. application of Viromer/FLuc complexes. Graphs displays floating bars (min to max) with line at mean. Two-way ANOVA, Sidak multiple comparison test. ***p < 0.001, n = 3. (**g**) Direct quantification of 7ND-mRNA (comparative) in different organs; Graphs displayed as box and whiskers plot (min to max). Line represents mean, n = 8.

Furthermore, the transfection of RAW264.7 cells by a Viromer/7ND (Fig. 1f) and Viromer/sTNF-RII-mRNA-complex (Fig. 1g) led to an accumulating concentration of both analytes in the cell culture medium. As the incubation time progressed, the concentration of 7ND and sTNF-RII increased significantly (7ND, **p < 0.01; sTNF-RII, ***p < 0.001). As such, an increase in 7ND and sTNF-RII protein followed an exponential growth rate up to the measured time point of 30 h (Supplementary Fig. 2c, d) suggesting substantial expression also between 24 and 30 h post-transfection. We did not observe an effect of Viromer complexes (100 ng mRNA/well) on RAW264.7 viability as determined by measuring intracellular LDH over the experimental time frame of 30 h (Supplementary Fig. 3.) In contrast, Chlorpromazine (100 µM), serving as toxicity control, clearly showed a negative effect on cell viability (Supplementary Fig. 3).

**Figure 3:**
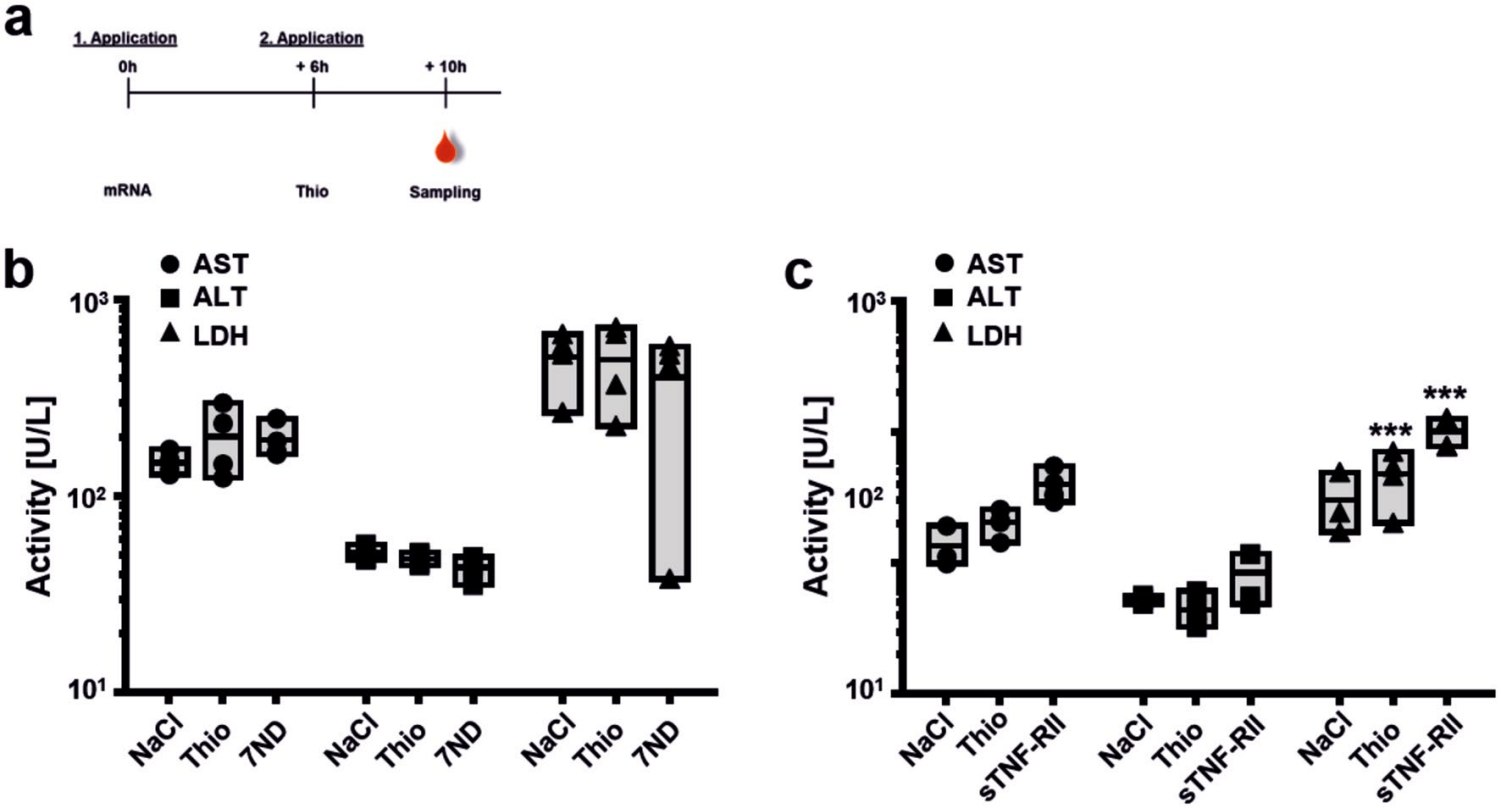
Liver and general toxicology in vivo. (**a**) Timeline for the analysis of liver (AST, ALT) and general (LDH) toxicology following 7ND-mRNA and sTNF-RII-mRNA application. (**b**,**c**) Activity of AST, ALT and LDH, 10 h after the i.v. application of (**b)** 7ND-mRNA (7ND) or (**c**) sTNF-RII-mRNA (sTNF-RII) in Thio-induced animals in comparison to control animals (NaCl-injection on both application time points) and Thio-induced animals without 7ND-application (Thio). Graphs displayed as floating bars (min to max) with line at mean. Two-way ANOVA, Sidak multiple comparison test. ***p < 0.001, n = 3–4.

In addition, no CCL2 induction was observed in untreated controls suggesting a silent transfection without induction of intrinsic CCL2 or sTNF-RII expression from these cells in vitro. This was further corroborated by application of Viromer RED and Viromer IN VIVO complexed with FLuc mRNA (100 ng/well) to RAW264.7 cells. Also, here no upregulation of cellular CCL2 was observed (Supplementary Fig. 3b).

Finally, since local concentrations of 7ND above 10–50 ng/ml would efficiently engage monocytes, the in vitro experiment suggested sufficient expression for an effect in vivo.

### Biodistribution after application of Viromer/FLuc-mRNA-complexes in vivo

After confirming the feasibility of transfection using Viromers in cell culture, the organ specificity of mRNA delivery was determined using the reporter FLuc-mRNA in complex with Viromer IN VIVO. The organ-specific transfection was monitored on the basis of two different application routes (i.v. and i.p.) in naïve mice, in addition to mice with induced peritonitis.

In a first in vivo imaging study the distribution of Viromer/Fluc complexes were monitored in living mice (Fig. 2a). Luciferase signals peaked 6 h after treatment, and most of the signals were detected in the spleen. Minor signals can be seen in the upper backbone, tail root, vertebrae and bones. The expression from FLuc mRNA is transient, with lower signals were detected after 24 h. In a follow-up study, the organ distribution of FLuc expression was studied following i.v. (Fig. 2b) and i.p. (Supplementary Fig. 4) application of Viromer/FLuc complexes. After 6 and 24 h, spleen, liver, PBMCs and bone marrow were isolated and FLuc expression was analysed. After 6 h, increased luciferase expression was detected in spleen, PBMC and bone marrow samples of the treatment groups. No or very low bioluminescence was detectable in liver samples as well as in spleen controls (Fig. 2b, Supplementary Fig. 4). FLuc expression in spleen was most pronounced and corroborated the findings from in vivo imaging. After 24 h, a reduced FLuc signal was detectable in samples underlining the transient nature of mRNA delivery. As a result, organ specificity of both tested routes was comparable. In addition, the blood cell count after 3 applications of Viromer IN VIVO/FLuc (30 µg) over a time frame of 9 days was determined in order to control acute effects of Viromer application on cellular blood composition (Supplementary Fig. 5a). There were no overt alterations in general blood parameters except for non-significant reductions of neutrophils and eosinophils (Supplementary Fig. 5b). There was no effect on monocytes as major cellular target population for following experiments.

**Figure 4:**
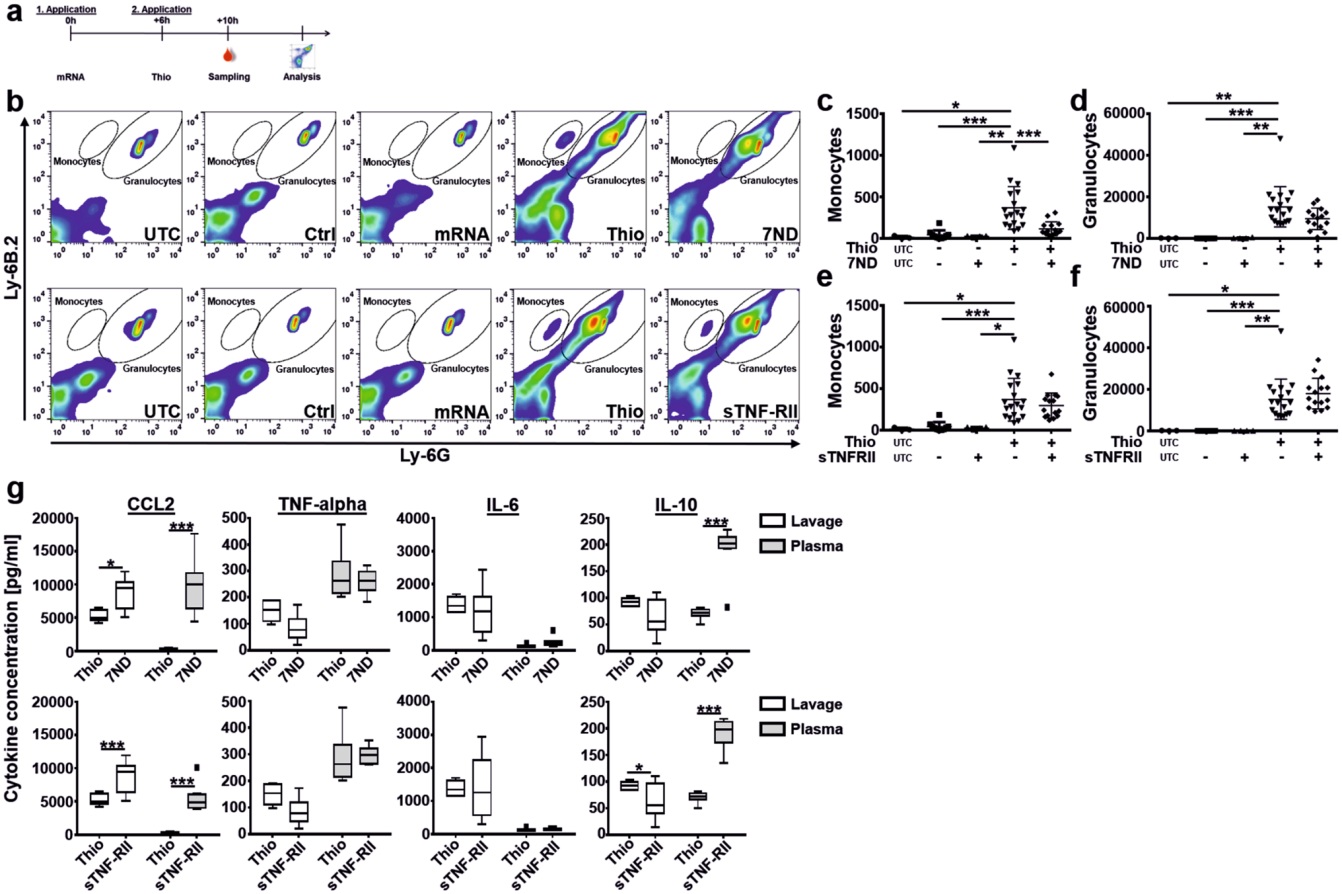
Efficacy of 7ND and sTNF-RII delivery on monocyte recruitment in the thioglycolate-induced peritonitis model. (**a**) Graphic illustration of experimental steps. (**b**) Representative FACS plots of each tested group. Gates for monocytes and granulocytes are indicated. Note, events in uninduced controls are caused by Trucount beads used for absolute quantification and were subtracted from the determined cell count. Illustrated are the groups UTC (untreated control), Ctrl (buffer injection in both time points), mRNA (Viromer/mRNA application without thioglycolate induction, Thio (thioglycolate induction) and 7ND/sTNF-RII (thioglycolate-induction with Viromer/mRNA treatment). (**c**–**f**) Number of infiltrating monocytes (**c**,**e**) and granulocytes (**d**,**f**) in the peritoneal lavage of the control groups without thioglycolate challenge in comparison to thioglycolate challenge and treatment using Viromer/7ND (**c**,**d**) and Viromer/s-TNF-RII (**e**,**f**). Mean ± SD, *p < 0.05, **p < 0.01, ***p < 0.001, one-way ANOVA, Tukey post-hoc test, n = 3 (UTC), n = 4 (mRNA), n = 10 (Ctrl), n = 15 (7ND and s-TNF-RII), n = 18 (Thio). (**g**) Bio-Plex analysis of the supernatant of the peritoneal lavage samples and plasma samples after *intravenous* administration of Viromer/7ND and Viromer/s-TNF-RII-mRNA-complexes. The concentrations of 4 cytokines (CCL2, TNF-alpha, IL-6 and IL-10) in plasma and peritoneal lavage samples were determined). Box and whiskers (Tukey), line at mean, *p < 0.05, ***p < 0.001, two-way ANOVA, Sidak multiple comparisons test, n = 7–8.

The biodistribution was also studied in the context of peritonitis for the i.v. route (Fig. 2c–g). The peritonitis model results in a substantial infiltration of monocytes (Fig. 2c, d) and granulocytes (Fig. 2c, e) 4 h after induction, accompanied by increased cytokine levels in lavage and plasma (Supplementary Fig. 6). The Viromer/FLuc complex was administered 6 h before thioglycolate-challenge and did not affect monocyte infiltration (Fig. 2d). The numbers of granulocytes were higher in FLuc-injected animals, however, lacking statistical significance when comparing the FLuc with the Thio groups (Fig. 2e). Peritoneal lavage and collection of spleen, liver, lavage cells and bone marrow were done 4 h later from all experimental animals. Increased luciferase expression in the samples of all analysed organs of the treatment group compared to the organ samples of the control group, could be detected (Fig. 2f). The relative expression level of FLuc was comparable to naïve animals and the strongest bioluminescence was again detected in the spleen samples.

A similar treatment scheme was used for analysis of cytokine induction in plasma after Viromer/FLuc application since nanoparticles could induce cytokine expression in vivo^20,21^. Here, mice received a NaCl or Viromer/FLuc injection 6 h prior to a second injection by NaCl. The second injection was used to simulate the second perforation of the peritoneum in a thioglycolate experiment. Analyses were done using cytokine arrays. Here, we could show, that CCL2 is found in higher amounts in plasma compared to naïve and NaCl-treated mice (Supplementary Fig. 7). We further quantified the concentration of CCL2 by ELISA and compared the plasma level to plasma CCL2 levels determined after application of thioglycolate w/o 7ND application. The ELISA results corroborated a slight, non-significant elevation of CCL2 after Viromer/FLuc application. Therefore, the application of the Viromers was not completely silent, and may also lead to the upregulation of additional cytokines besides CCL2 and the other studied 21 cytokines (Supplementary Fig. 7). This, in turn could account for a slightly higher granulocyte infiltration in FLuc-injected animals (Fig. 2e), however only 7ND application leads to substantial CCL2 levels in plasma, supporting the conclusion of 7ND expression in vivo (Supplementary Fig. 8).

Finally, the organ distribution of 7ND-mRNA was directly analysed in the context of peritonitis induction (Fig. 2g). This was possible since the applied 7ND mRNA differs at the N-terminus from native murine CCL2 by a deletion of codons, coding for amino acids 2–8. No such experiment was performed for sTNF-RII. The Viromer/7ND complex was administered i.v. and analysis was done in spleen, liver, cells from the peritoneal lavage, plasma and bone marrow. In all samples of the treatment group (application of the Viromer/7ND-mRNA complex) an increased amount of 7ND-mRNA was detectable compared with controls. Interestingly, in contrast to the FLuc determinations, bone marrow demonstrated the highest concentration of 7ND-mRNA (Fig. 2g).

### Identification of a suitable application route, analysis of liver and general toxicology

In a pilot experiment to study possible differences in cellular infiltration depending of the application route, Viromer/7ND and Viromer/sTNF-RII were applied i.v. (Supplementary Fig. 9a–e) or i.p. (Supplementary Fig. 9f–j), in the context of induced peritonitis. Due to the transient nature of protein expression after FLuc-mRNA delivery (Fig. 2), mRNAs were administered 6 h before peritonitis was triggered by the application of thioglycolate, in order to ensure high therapeutic protein levels. After 4 h, peritoneal lavage was performed and the obtained cellular infiltrate was analysed by FACS (Supplementary Fig. 9a, f).

As expected, the number of infiltrating granulocytes exceeded the number of infiltrating monocytes (e.g. Supplementary Fig. 9b vs. Supplementary Fig. 9c) by at least one order of magnitude. When comparing the cellular infiltration among test groups, it was noticeable that the infiltration of monocytes and granulocytes was much higher in the treatment groups (7ND-mRNA and sTNF-RII) after i.p. application than in thioglycolate-induced controls (Supplementary Fig. 9f–j).

In contrast, a reduced infiltration of monocytes was visible after application of 7ND and sTNF-RII via the i.v. route compared to thioglycolate application alone (Supplementary Fig. 9b, c). Obviously, the complex boosted the cellular infiltration into the peritoneum after i.p. application (Supplementary Fig. 9f–j). Therefore, the i.p. route of Viromer application was excluded from further testing.

As an additional control, toxicity of 7ND (Fig. 3a, b) and sTNF-RII (Fig. 3a, c) was determined for the prioritized i.v. application route. The activity levels in plasma of liver-specific enzymes ALT and AST as well as the general toxicity marker LDH was analysed and used as decisive values for the assessment of toxicity in vivo.

As a result, there was no overt toxicity of acute Viromer/-mRNA-complex treatment, except for a non-significant approx. two-fold increase in AST, and a significant four-fold increase of LDH after sTNF-RII application (Fig. 3c). For 7ND application, the enzyme activities of ALT and LDH from the treatment groups were comparable to those of the control groups and those of the untreated animal (Fig. 3b). Overall, the acute treatment was safe and experiments were progressed to an in vivo proof of concept.

### Effectivity of mRNA delivery in the model of thioglycolate-induced peritonitis

As described earlier, the administration of mRNAs was 6 h before thioglycolate challenge in order to ensure highest therapeutic protein expression at the time of peritonitis induction (Fig. 4a). Again, the induction of peritonitis resulted in a substantial cellular infiltration (Fig. 4b), which was intended to determine whether the in vitro results led to a meaningful concentration of 7ND and/or sTNF-RII in vivo, in the context of altering cellular infiltration. The injection of Viromer/mRNA-complexes was tested in comparison to control groups. Application of 7ND-mRNA had a pronounced effect on monocyte infiltration into the peritoneum (Fig. 4b, c). In contrast, the infiltration of granulocytes (Fig. 4d) was only slightly, and non-significantly, affected suggesting a specific and expected influence of 7ND on the CCL2/CCR2 axis. A trend of reduced monocytes after application of sTNF-RII, as seen in the pilot experiment (Supplementary Fig. 9d), was also observed here. However, the lower mean did not reach statistical significance (Fig. 4e). Granulocyte infiltration was not affected by sTNF-RII application (Fig. 4f). Finally, the concentration of inflammatory cytokines in samples with thioglycolate challenge were determined by Bio-Plex analysis (Fig. 4g). Depicted are the measurements for CCL2, TNF-alpha, IL-6 and IL-10.

CCL2 and TNF-alpha were chosen since they are directly involved in the applied treatment. IL-6 and IL-10 are also analysed, since these cytokines showed a regulation among groups. As depicted, the samples of peritoneal lavage and plasma revealed an increased CCL2 concentration after expression of 7ND and sTNF-RII compared with the group that received thioglycolate alone. The increase after 7ND administration results from a combination of intrinsic murine CCL2 induction by Viromer IN VIVO application, and additional 7ND expression since Viromer/FLuc also resulted in a non-significant increase of plasma CCL2 (Supplementary Fig. 8). Viromer/sTNF-RII administration also led to an increased concentration of CCL2, although the measured levels were lower compared to the expression of 7ND. Therefore, the combination of Viromer and sTNF-RII is able to induce CCL2, although the exact mechanism awaits further elucidation (Fig. 4g).

The concentration of IL-10 and TNF-α in lavage samples appeared to be reduced in the 7ND-mRNA and sTNF-RII-mRNA treatment groups compared with the control groups. The IL-6 concentration in the lavage fluid was comparable in all groups.

Plasma measurement showed a strong increase of IL-10 compared to controls after expression of 7ND and sTNF-RII, suggesting an anti-inflammatory reaction. In contrast, the readings for IL-6 and TNF-α indicated comparable concentrations in the plasma of the control group and the 7ND-mRNA treatment group.

## Discussion

In the present study we have shown that Viromers, recently-developed carriers for nucleic acids, are able to efficiently deliver therapeutic mRNAs, and mediate their biologic effect in vivo. The concept of mRNA-based therapeutics has received considerable attention, especially in the fields of vaccination^22,23^, cancer immunotherapies^5,24^ and protein replacement therapies, e.g. in oncology^8,25,26^ and reprogramming of immune effector cells^9^.

A further therapeutic option for mRNA delivery that is supported by our present study, is the expression of experimental or approved biologic molecules directly in the patient in relation to the field of inflammation.

In this regard, we analysed the potency of 7ND and sTNF-RII to alleviate cellular infiltration in a model of thioglycolate-induced peritonitis. The model is appropriate to study CCL2-mediated monocyte-infiltration^18^ and chemokine-induced granulocyte infiltration^27^. In addition, previous studies have already explored the suitability of chemokine expression in monocytes, including native CCL2 after delivery using Viromer RED^9^.

Both applied biological molecules have been extensively studied in the context of suppressing inflammatory disorders. 7ND was successfully tested in a number of preclinical and CCL2-related animal models, such as atherosclerosis and restenosis^28,29^ or renal fibrosis^30^. Additionally, neutralization of TNF-alpha by application of an engineered fusion protein of the soluble ligand binding domain of TNF-RII and a F_C_-subunit of human IgG1 is approved for the treatment of rheumatoid arthritis^31^ and psoriasis^32^.

The infiltration of immune cells to the peritoneum correlates with the presence of specific chemokines^33^, which are produced, e.g. by mesothelial cells^34^. Also, for human peritoneal fibroblasts, it was shown that these cells are capable of generating CCL2 and TNF-alpha, depending on NFκB-induction. Furthermore, TNF-alpha is able to augment CCL2 expression in these cells^35^. Both strategies to prevent monocyte infiltration into the peritoneal cavity by interfering with CCL2 action^18,36^ or TNF-alpha function^37^, have been successfully applied in the thioglycolate model.

Based on these parameters, we expected a direct effect on cellular infiltration by expressing 7ND and a general immunosuppressive effect by neutralizing the activity of TNF-alpha, with the effect of 7ND likely to be more pronounced than the effect of TNF-alpha neutralization, since it has been previously shown that neutralizing CCL2 greatly diminishes monocyte-infiltration in this model^18^, whereas, the effect, e.g. of methotrexate application is rather mild^37^. Indeed, we were able to show a significant effect of 7ND expression on monocyte-infiltration, whereas sTNF-RII application showed only a trend of reduced cellular infiltration. Therefore, the achieved concentrations after delivery of 7ND-mRNA in vivo were sufficient to block monocyte infiltration.

The observed effect was strongly dependent on the route of application. While successful treatment was seen after intravenous application of the Viromer/7ND complex, the intraperitoneal route aggravated cellular infiltration irrespective of the applied mRNA, and might therefore, be related to the applied Viromers. This assumption was supported by application of Viromer/FLuc complexes specifically increasing the concentration of CCL2 in plasma, whereas, other screened cytokines did not show a difference. Therefore, the delivery of Viromer/mRNA complexes to phagocytes in conjunction with a second inflammatory stimulus (thioglycolate) may trigger an inflammatory cascade^38^ that aggravates inflammation in the applied peritonitis model. From a translational aspect, the i.p. route is not used in humans, however further evaluation of Viromers are suggested by this study.

Due to the pronounced differences of application routes, intravenous application was chosen for further experiments. This was straightforward, since the targeted organs after application the Viromer/mRNA complexes did not differ between the tested routes. Spleen cells and bone marrow were the primary sites of target mRNA expression.

Furthermore, differences in observed potencies of 7ND and sTNF-RII could also result from the primary site of antagonistic action of the tested biomolecules. As stated, CCL2 is generated by different cell types within the peritoneum after thioglycolate challenge. It is then bound to glycosaminoglycans in the subendothelial space^39^ and on endothelial cells, subsequently attracting monocytes from the circulation and guiding these cells through a chemotactic gradient^40^ across the mesothelial lining and into the peritoneal cavity. Therefore, since 7ND is produced, e.g. by spleen cells after gene transfer, it will be present in the circulation and bind to CCR2 without receptor activation^41^. Consequently, if CCR2 is blocked, circulating monocytes will not recognize the CCL2 gradient leading to the peritoneum.

In contrast, TNF-alpha was shown to increase CCL2 expression of peritoneal fibroblasts^35^. Therefore, its primary site of action lies within the peritoneal cavity and sTNF-RII molecules expressed, e.g. by spleen cells need to enter the peritoneal space. This was not the case in the tested model. Therefore, further testing of sTNF-RII would require a model better suitable for studying the effect of sTNF-RII delivery, such as collagen-induced arthritis in mice.

Nevertheless, we were able to achieve proof-of-concept for the expression of anti-inflammatory biologics mediated by Viromer delivery. This might open novel perspectives concerning the application of such therapeutic molecules in the future. Currently, biologics are supplemented by biosimilar molecules after patent protection ends. While these molecules introduce competition to the market, which usually leads to lower pricing, each molecule has still a unique manufacturing process and specific properties, resulting from the applied production line and may contain modifications of protein structure, function and pharmacology that could differ from the original product. These modifications are, expected by medical agencies, to be identified and handled by the manufacturer^42,43^. Therefore, shifting the required protein production of biologics into the patient rather than costly production in foreign hosts with the necessity of extensive characterization, could be a future alternative, that might substantially reduce costs for such molecules, and would make them available to a greater patient population, in particular those in less developed countries.

In summary, we have shown, that gene therapy by application of mRNAs coding for anti-inflammatory biological molecules is feasible. The observed effect, especially for 7ND application, was similar to what would be expected after application of an, e.g. neutralizing antibody. Therefore, Viromers could help to efficiently deliver mRNA molecules in patients with a wide range of application possibilities. The use of translational and more sophisticated animal models will help to further qualify Viromers as suitable carrier system under inflammatory conditions.

## Methods

### Preparation of Viromer and mRNA

Viromer-RED (Lipocalyx) was used for in vitro transfection experiments of RAW 246.7 cells according to the manufacturer’s instructions. Viromer mRNA IN VIVO is a lyophilized reagent containing pre-formed nanoparticles (provided by Lipocalyx). The mRNA IN VIVO reagent was used for all animal experiments.

The applied mRNAs were synthesized by TriLink Biotechnologies (San Diego, CA, USA) and are silica gel purified. The applied firefly-luciferase-mRNA (FLuc, cat# L-7202) had 1921 nucleotides, the EGFP-mRNA consisted of 996 nucleotides (cat# L-7201). Both reporter gene mRNAs are fully substituted with 5-methoxyuridine and capped using CleanCap (Cap 1 structure).

The mRNA coding for murine 7ND consisted of 702 nucleotides and the mRNA encoding murine sTNF-RII possessed 1,050 nucleotides. Both target mRNAs are fully substituted with 5-methylcytidine and capped using CleanCap (Cap 1 structure).

The mRNA stock solutions (7ND mRNA: 1.21 mg/ml; sTNF-RII: 1.43 mg/ml; GFP-mRNA: 1 mg/ml; FLuc: 1 mg/ml) were diluted to 400 µg/ml using RNAse-free water (Lonza). Before application to mice, Viromer mRNA IN VIVO was rehydrated using the diluted mRNA stock solution.

For the transfection of the RAW 246.7 cells, all mRNAs were prepared by diluting the corresponding mRNA stock solution to 11 ng/μl in Viromer-RED-buffer. Viromer solution was prepared according to manufacturer’s recommendations. Briefly, Viromer-RED was diluted 1:25 with Viromer-RED-buffer and vortexed 5 s. To form the Viromer/mRNA-complex, 45 μl of the mRNA dilution was mixed with 5 μl of the Viromer solution, homogenized and incubated for 15 min at room temperature. Subsequently, 10 μl of the Viromer/mRNA-complex per 96-well were added to the cells. One hundred (100) ng mRNA/well have been used in cell culture experiments.

### Particle size determination

The particle size of Viromer/mRNA complexes was measured non-diluted by dynamic light scattering (DLS) using a 3,000 HSA Zetasizer from Malvern Instruments Ltd. Average particle size is recorded as Z_average_ value and size distribution (polydispersity index, PDI) was calculated in the multimodal mode.

### Determination of Zeta potential (surface charge)

The Zeta potential of Viromer/mRNA complexes was determined by laser Doppler electrophoresis (LDE) using a 3,000 HSA Zetasizer from Malvern Instruments Ltd. Complexes were diluted in Hepes/NaOH-Sucrose, pH 7.4 to a final RNA concentration of 10 µg/ml. The Zeta potentials were averaged for three single, independent measurements.

### Determination of accessible (non-bound) mRNA

RiboGreen assay: the Quant-iT RiboGreen RNA assay Kit (ThermoFisher Scientific/Life Technologies; cat# R11490) was used according to the manufacturer’s instructions. The mRNA was diluted to 5 µg/ml in TE buffer. For standard curve the mRNAs were serially diluted to 0.04 µg/ml. Samples were diluted in TE-buffer to 2.5 µg/ml and the RiboGreen reagent was added accordingly. Fluorescence signals were measured in a plate reader at Ex485nm/Em505nm.

### Cell culture and transfection of RAW 264.7 cells

For routine cell culture, 4 × 10^6^ cells were seeded in 75 cm^2^ cell culture flasks (Greiner) containing 15 ml cell culture medium (Dulbecco's Modified Eagle Medium (DMEM); Life Technologies GmbH) supplemented with 10% foetal bovine serum ((FBS); Life Technologies GmbH). Cell were grown at 37 °C and 5% CO_2_. For passaging, cells were detached from the plastic surface with 3 ml of trypsin-EDTA solution (0,05% trypsin, 0,02% EDTA (Life Technologies GmbH, Germany)). Determination of cell counts was done using a cell counter (CASY, OMNI Life Science). Cells were used until passage 15 after thawing.

For transfection experiments, 5 × 10^4^ living cells per well were seeded in a 96-well cell culture plate (Greiner) containing 100 μl of cell culture medium. Cells were incubated for 24 h at 37 °C and 5% CO_2_. At the day of transfection, 10 μl of the Viromer/mRNA-complex per 96-well were added to the cells. After 6 h, 24 h and 30 h cell culture supernatants were collected for analysis of CCL2 and sTNF-RII expression. The same timepoints were used for analysis of cellular expression of GFP and FLuc.

### Cellular viability assay

Cell viability after Viromer/mRNA application was assessed using RAW264.7 cells plated in 96-well microtiter plates (Greiner bio-one) at a density of 50.000 cells/well. After 24 h, Viromer/mRNA complexes (100 ng mRNA/well) were added to fresh medium and compared to the application of Viromer buffer and 100 µM Chlorpromazine (in 1% DMSO). Incubation was for 6 h, 24 h and 30 h. Cellular viability was determined using the CytoTox-ONE kit (Promega). For intracellular LDH analysis, cells were washed 2 times with PBS, lysed using 9% Triton-X100 in PBS for 10 min. Afterwards, substrate mix was added to the wells and the reaction was stopped after 10 min. Fluorescence was determined using a CLARIOstar plate reader (BMG Labtech) at ex/em 544/595 nm.

### ELISA

For the determination of the CCL2/7ND and sTNF-RII expression, commercially available mouse Platinum CCL2-ELISA (Invitrogen) and mouse sTNF-RII-ELISA (RayBiotech) were used. Notably, the CCL2-ELISA also detected 7ND. Both ELISA were performed according to the provided manuals. The absorption measurement was carried out using a Spectra-Fluor Plus plate reader (Tecan Group) at a wavelength of 450 nm and a reference wavelength of 570 nm with the aid of the device software XFluor4 (Tecan Group).

### Cytokine arrays

Mouse cytokine antibody array C1 (RayBiotech) was performed according to the manufacturers recommendations using murine plasma. The membranes were developed using SuperSignal West Pico (ThermoFisher) as substrate for HRP. Quantification was done using ImageJ software. Blank was subtracted from all samples and dot intensities were related to the intensity of the positive control and expressed as relative expression compared to positive control of the arrays.

### Animals

For all experiments, male mice of strain DBA/1 purchased from Janvier Labs were used. Young adults from an age of 8 weeks up to a maximum age of 30 weeks were used for the experiments. The in-house experiments using DBA/1 were approved by the responsible animal ethics committee of the state of Saxony-Anhalt, Germany (Landesverwaltungsamt Sachsen-Anhalt, Department of Consumer Protection and Veterinary Affairs, Halle (Saale), Saxony-Anhalt, Germany) under the following approval number: 42502-2-1472 MLU. We confirm that all animal handling procedures were carried out in accordance with directive 2010/63/EU of the European Parliament and of the Council on the protection of animals used for scientific purposes, the German Animal Protection Act and recommendations from the Federation of European Laboratory Animal Science Associations (FELASA).

Throughout the experiment, the mice were housed in individually ventilated cages (IVC, type II long, Tecniplast) with 5 mice per cage. The standardized housing conditions (room temperature: 22 °C ± 2 °C, humidity: 50% ± 20%) were controlled by a central ventilation system. The regulation of the day-night-rhythm (12 h day/12 h night) took place via a timer. The animals were given standard mouse food (ssniff: mouse breeding) and tap water with drinking water quality ad libitum.

### In vivo imaging

Female hairless mice of strain Crl:SKH1-Hr^hr^ (Charles River Laboratories) of an age of 7 weeks were used for in vivo imaging. Studies were conducted by an external CRO (Preclinics GmbH, Potsdam, Germany). First, the mice were put under infrared light for vasodilation. 30 µg of Viromer-complexed FLuc mRNA imaging, the tail vein was punctured using a 27G cannula for luciferin administration. Injection volume was 10 mL/kg containing 15 mg/mL luciferin. Afterwards the animals were anesthetized isoflurane inhalation. The animals were imaged from the ventral side for 300 s under continuous isoflurane anaesthesia. Then the animals were turned and imaged again for 300 s from the lateral side. Expression of luciferase was monitored after 6 and 24 h using a luminescence CCD camera (Andor Technology Ltd). Afterwards, animals were allowed to wake up.

### Differential blood count

Effect of repeated mRNA application in vivo was analysed by applying 30 µg FLuc mRNA in Viromer IN VIVO to female hairless mice of strain Crl:SKH1-Hr^hr^ (Charles River Laboratories). Applications (i.v.) were done on day 1, 4 and 8 followed by determination of differential blood count on day 9. For this, the animals were anesthetized with 5 vol% isoflurane and whole blood of the unconscious animal was removed by retro-orbital bleeding. The blood was collected in 1.3 ml-lithium-heparin microtubes. 120 µl of whole blood was used for measurement of the differential blood count using the Cell-Dyn 3700 (Abbott, IL, USA) according to the instruments instruction. The study was conducted by an external CRO (Preclinics GmbH, Potsdam, Germany).

### Thioglycolate-induced peritonitis and peritoneal lavage

For induction of peritonitis, an 8% thioglycolate solution was prepared. For this, 8 g of thioglycolate (Sigma-Aldrich) were resuspended in 100 ml of a 0.9% NaCl solution (Covetrus). The suspension was autoclaved at 122 °C for 40 min to dissolve the thioglycolate powder. Afterwards, the solution was stored at 8 °C^18^. The first application (intravenous (i.v.) or intraperitoneally (i.p.)) was in dependence of the treatment group. Mice received either 150 μl Viromer-buffer or 150 µl Viromer-mRNA-complex (60 µg). The application of 2 mg/kg body weight (BW) thioglycolate (8% solution in NaCl) or an equivalent volume of NaCl was 6 h later by i.p. injection.

Another 4 h later, all animals were anesthetized using isoflurane (CP-pharma) and 8 ml of PBS (Life Technologies GmbH) were applied i.p. for peritoneal lavage. By abdominal massage, the PBS distributed evenly in the abdomen and approx. 4–5 ml of lavage fluid were obtained and transferred to a 15 ml tube.

The painless killing of the animals was carried out after the peritoneal lavage by cardiac puncture. The cellular composition in the lavage fluid was analysed by FACS.

### Blood and organ collection

Blood samples obtained by cardiac puncture were transferred to lithium-heparin tubes (Sarstedt) and centrifuged at 2000×*g* for 10 min at 4 °C. Thereafter, the plasma was transferred to a 0.5 ml reaction tube, snap frozen on dry ice and stored at − 80 °C. For bone marrow removal, both hind legs were cut and after transection of the femur and tibia, the ossa femora were removed. The opened bone marrow cavity was punctured using a 23 G cannula and the bone marrow was rinsed with 1 ml of ice-cold Hank's balanced salt solution (HBSS (Life Technologies GmbH)) and collected in a 1.5 ml reaction tube. The bone marrow-HBSS solution was further transferred to a 15 ml Falcon tube, filled-up to 15 ml with HBSS and homogenized by mixing with a single-channel pipette. After centrifugation at 200×*g* for 8 min, the supernatants were discarded and the cell pellets resuspended in 10 ml of erythrocyte lysis buffer. Following an incubation for 2 min at RT, the samples were again centrifuged at 200×*g* for 8 min. The supernatants were discarded and the pellets were resuspended in 1 ml HBSS. Afterwards, the suspensions were transferred to 1.5 ml reaction tubes and centrifuged at 200×*g* for 8 min. The resulting cell pellets were stored on ice until immediate bioluminescence analysis.

For the perfusion of the bloodstream, a blunt 21 G cannula was inserted through the right ventricle into the aorta after blood extraction and the blood vessels were perfused with 20 ml PBS. Subsequently, the spleen and the right liver lobe (*lobus hepatis dexter*) were cut at the hilum, transferred to an empty 1.5 ml reaction tube and snap frozen on dry ice. The organs were stored at − 80 °C.

### FACS analysis

GFP and FLuc expression in the harvested cells from the cell culture experiments were analysed by FACS. For this, the cells were transferred into 300 μl FACS buffer (PBS+3% FBS). The GFP samples were analysed directly thereafter.

The FLuc cells were first resuspend in 100 μl 4% paraformaldehyde (Life Technologies GmbH). Following incubation for 20 min at RT, the cells were washed once with 3 ml PBS/BSA (Life Technologies GmbH). In the next step the cells were resuspend in 100 µl 0.1% saponin and incubated for 15 min at RT. After that the cells were incubated with the primary antibody, anti-luciferase (Bio-Rad Antibodies) and incubated for 30 min at 4 °C in the dark. This was followed by three times washing with 1 ml 0.1% saponin and a resuspension with 100 µl 0.1% saponin. Then the secondary antibody anti-IgG1-FITC (Miltenyi Biotec) was added, which also served as isotype control, and incubated 30 min at 4 °C in the dark. Following one time washing with 3 ml 0.1% saponin, the cell pellets were resuspended in 200 µl 0.5% paraformaldehyde an analysed by FACS.

Cells in lavage fluids were harvested by centrifugation at 300×*g* for 5 min. Visible contaminations with erythrocytes were removed by the addition of 10 ml erythrocyte lysis buffer (155 mM NH_4_Cl; 10 mM KHCO_3_; 126 µM EDTA in H_2_O bidest.) as preliminary experiments have shown that these interfere with the FACS analysis. To prevent non-specific binding of the antibodies to Fc-receptors, all samples were incubated with a CD16/CD32 Fc-blocker (Life Technologies GmbH) prior to staining using specific antibodies. The isotype controls were rat IgG2a conjugated to Alexa-Fluor 647 (Bio-Rad Antibodies) and PerCP-Vio 700 (Miltenyi Biotec), respectively. Two different fluorescently labelled antibodies were used to separate infiltrating cells: Ly-6B.2-Alexa Fluor 647 (Bio-Rad Antibodies) and Ly-6G-PerCP-Vio700 (Miltenyi Biotec). The preparation of the samples was based on the manufacturer's instructions for the applied antibodies. Following centrifugation at 400×*g* for 5 min, the cell pellets were washed with 10 ml PBS and centrifuged again at 400×*g* for 5 min. The supernatant was discarded and the cell pellets resuspended in 1 ml of FACS buffer. All FACS tubes were incubated with the respective antibodies for 10 min at 4 °C in the dark. After incubation, cells were washed with 1 ml PBS followed by centrifugation at 400×*g* for 5 min. The supernatant was discarded and the cell pellet was resuspended in 200 μl of FACS buffer. Subsequently, cells in a total volume of 200 μl of FACS buffer were transferred to a counting beads-containing Trucount tube (BD Bioscience) and incubated for 30 min at room temperature. Prior to flow cytometry, the cells and beads were vortexed. FACS analysis was done using FACSCalibur (BD Bioscience) with appropriate settings. Analysis was done using FlowJo version 10 (FlowJo LLC).

### Biodistribution of firefly-luciferase (FLuc)-mRNA

The experiments for the biodistribution of FLuc mRNA in vivo were carried out in 3 separate test sections. In the first experiment, one group received an i.p. application of 150 μl PBS. The other group received an i.p. application of 150 μl of the Viromer/FLuc-mRNA-complex. In the second experiment, the i.v. application was analysed accordingly. Half of the animals in each group were anesthetized with isoflurane six hours after application, the other half 24 h after application, and the liver, spleen, bone marrow and blood were collected.

In the third part of the experiment, the test animals were again divided into 2 groups. Both groups initially received an i.v. administration, depending on the treatment group either with 150 μl PBS or Viromer/FLuc-mRNA-complex. Six hours later, all animals received an i.p. application of 2 g/kg BW 8% thioglycolate solution. 4 h after the i.p. application all animals were anesthetized with isoflurane and liver, spleen, bone marrow and plasma were collected and peritoneal lavages was performed.

### Isolation of hepatocytes and splenocytes

The isolation of hepatocytes was carried out from pre-treated pieces of collected livers^44^. Splenocytes were isolated from crushed pieces of spleen incubated in 40 ml of a digestive buffer (Collagenase IV: 1 mg/L; DNase I: 50 mg/L; FBS: 1% (v/v); PBS) for 45 min at 37 °C. For both cell types, the respective mixtures were first passed through a 100 μm cell strainer, followed by a passage using a 40 μm cell strainer. Afterwards, the samples were centrifuged at 300×*g* for 10 min at 4 °C. The supernatants were discarded and the cell pellets were incubated in 10 ml of erythrocyte lysis buffer at room temperature. Following centrifugation at 300×*g* for 10 min at 4 °C, the cell pellets were transferred to 1.5 ml reaction tubes and stored on ice until bioluminescence analysis.

### Isolation of PBMCs by gradient centrifugation

To isolate peripheral blood mononuclear cells (PBMCs) from the recovered blood samples, gradient centrifugation was performed using Percoll^45^. In order to adjust the Percoll solution to a density of 1.078 g/ml, 11.11 ml Percoll, 2 ml 1.5 M NaCl and 6.89 ml H_2_O dest. were mixed for a 20 ml batch. The collected blood from all animals of one experimental group was pooled in a 15 ml Falcon tube and mixed with PBS to a total volume of 7 ml. Subsequently, the mixture was added to 3 ml of the Percoll dilution placed in a 15 ml Falcon tube and centrifuged at 900×*g* for 30 min without brakes. The resulting interphase was transferred to a new 15 ml Falcon tube and mixed with HBSS to a total volume of 10 ml.

Following centrifugation at 400×*g* for 10 min, the supernatants were aspirated, the cell pellets resuspended in 10 ml of erythrocyte lysis buffer and incubated for 2 min at RT and centrifuged again at 400×*g* for 5 min. In case the cell pellets still had a red colour, the lysis step was repeated. Thereafter, the cell pellets were transferred to 1.5 ml reaction tubes and stored on ice until immediate bioluminescence analysis.

### Bioluminescence analysis

For analysis of FLuc bioluminescence, cell pellets were brought to RT. Depending on the cell number, 10–25 μl of the cell pellet were lysed using 150 μl of 1 × Beetle Lysis Juice. The samples were incubated for 10 min in the dark at RT. Thereafter, 100 μl of the solution were transferred to a white 96-well plate. The bioluminescence of each well was measured at one second integration time on the Glomax plate reader (Promega).

### Direct quantification of 7ND mRNA

Organs (liver, spleen, bone marrow, blood cells) were directly analysed for the presence of 7ND-mRNA by an external CRO (Axolabs, Kulmbach, Germany). For the specific quantification of the mRNA, the branched DNA technology (QuantiGene) was used. The method is based on the cooperative hybridization of target mRNA molecules and a gene-specific sample set (single-stranded DNA sequences). This sample set consists of 6 Capture Extender DNA sequences (hybridization of the 5′ end with target mRNA, the 3′ end with single stranded capture DNA sequences (immobilized on assay plate)), 14 label extender DNA sequences (hybridization of the 5′ end with the target mRNA, the 3′ end provides the basis for hybridization with the 2.0 PreAmplifier) and 4 blocking probes. The number of label extender sequences defines the sensitivity of the assay because the amplifiers coupled with label probes (luminescent signal) bind to it via the hybridization of the pre-amplifier in the subsequent step. Since the luminescence signal is proportional to the amount of target mRNA, the mRNA content can be determined via the luminescence measurement considering a reference gene (glyceraldehyde-3-phosphate dehydrogenase, (GAPDH)).

### Toxicity in vivo by determination of AST, ALT and LDH

For determining liver toxicity (AST, ALT) and general toxicity (LDH), 300 μl of blood were taken from the experimental animals during the final cardiac puncture and transferred to lithium-heparin tubes. The tubes were centrifuged for 10 min at 2000×*g* and 4 °C and approx. 90 µl of plasma were transferred to 0.5 ml reaction tubes. The samples were analysed by central laboratory of the Faculty of Veterinary Medicine (University of Leipzig, Germany), using laboratory automats for the determination of clinical-chemical parameters (COBAS c311, Roche).

### Bio-Plex analysis

For multiplex analysis of plasma and peritoneal lavage samples an individually prepared 9-Plex (Bio-Rad) was used. The following cytokines were included: IL-1β, IL-6; IL-10; MIP-1α, MIP-1β, IFN-γ, RANTES, CCL2 and TNF-α. At the beginning, all desired cytokine beads were combined and each 100 µl of the suspension was added per well to a 96-well plate. After washing twice, the samples (1:4 plasma and 1:2 lavage fluid diluted in Sample Diluent), standards and blanks were transferred to the appropriate wells. Further steps of the implementation were made according to the manufacturer's instructions.

### Statistical analysis

Results were analysed using GraphPad Prism version 6.0 for Windows (GraphPad Software). Depending on the data set, one-way or two-way ANOVA was performed. This was followed by post-tests. Dunnett test was used after one-way ANOVA, when samples were compared to a control sample. In addition, Tukey or Sidak multiple comparison test was used after one-way ANOVA for analysing all columns. Finally, Sidak test was used after two-way ANOVA. Significance levels are illustrated by asterisk. A *p* value < 0.05 was considered significant.

## Supplementary information

Supplementary Information.

## References

[1] Moreland LW (1997). Treatment of rheumatoid arthritis with a recombinant human tumor necrosis factor receptor (p75)-Fc fusion protein. N. Engl. J. Med..

[2] Marziniak M, Meuth S (2014). Current perspectives on interferon Beta-1b for the treatment of multiple sclerosis. Adv. ther..

[3] Mountain A (2000). Gene therapy: the first decade. Trends Biotechnol..

[4] Hallek M (2001). Grundlagen der Gentherapie. Prinzipien und Stand der Entwicklung. Der Internist.

[5] Sahin U, Karikó K, Türeci Ö (2014). mRNA-based therapeutics: developing a new class of drugs. Nat. Rev. Drug Discov..

[6] Kranz LM (2016). Systemic RNA delivery to dendritic cells exploits antiviral defence for cancer immunotherapy. Nature.

[7] Cedervall T (2007). Understanding the nanoparticle-protein corona using methods to quantify exchange rates and affinities of proteins for nanoparticles. Proc. Natl. Acad. Sci..

[8] Fiore A (2018). Induction of immunosuppressive functions and NF-κB by FLIP in monocytes. Nat Commun..

[9] Xu Y (2019). Exploitation of synthetic mRNA to drive immune effector cell recruitment and functional reprogramming in vivo. J. Immunol. (Baltim. Md. 1950).

[10] Panzner S (2014). Transfection: viral and synthetic techniques converge. Gen. Eng. Biotechnol. News.

[11] Rao S, Morales AA, Pearse DD (2015). The comparative utility of Viromer RED and lipofectamine for transient gene introduction into glial cells. BioMed Res. Int..

[12] Usui M (2002). Anti-monocyte chemoattractant protein-1 gene therapy inhibits restenotic changes (neointimal hyperplasia) after balloon injury in rats and monkeys. FASEB J..

[13] Zhang YJ, Rutledge BJ, Rollins BJ (1994). Structure/activity analysis of human monocyte chemoattractant protein-1 (MCP-1) by mutagenesis. Identification of a mutated protein that inhibits MCP-1-mediated monocyte. J. Biol. Chem..

[14] Zhang Y, Rollins BJ (1995). A dominant negative inhibitor indicates that monocyte chemoattractant protein 1 functions as a dimer. Mol. Cell Biol..

[15] Gong JH, Ratkay LG, Waterfield JD, Clark-Lewis I (1997). An antagonist of monocyte chemoattractant protein 1 (MCP-1) inhibits arthritis in the MRL-lpr mouse model. J. Exp. Med..

[16] Gong JH, Clark-Lewis I (1995). Antagonists of monocyte chemoattractant protein 1 identified by modification of functionally critical NH2-terminal residues. J. Exp. Med..

[17] Gu L (1998). Absence of monocyte chemoattractant protein-1 reduces atherosclerosis in low density lipoprotein receptor-deficient mice. Mol. Cell.

[18] Cynis H (2011). The isoenzyme of glutaminyl cyclase is an important regulator of monocyte infiltration under inflammatory conditions. EMBO Mol. Med..

[19] Takahashi M, Galligan C, Tessarollo L, Yoshimura T (2009). Monocyte chemoattractant protein-1 (MCP-1), not MCP-3, is the primary chemokine required for monocyte recruitment in mouse peritonitis induced with thioglycollate or zymosan A. J. Immunol..

[20] Maugeri M (2019). Linkage between endosomal escape of LNP-mRNA and loading into EVs for transport to other cells. Nat. Commun..

[21] Kumar V (2014). Shielding of lipid nanoparticles for siRNA delivery: impact on physicochemical properties, cytokine induction, and efficacy. Mol. Ther. Nucleic Acids.

[22] Petsch B (2012). Protective efficacy of in vitro synthesized, specific mRNA vaccines against influenza A virus infection. Nat. Biotechnol..

[23] Brazzoli M (2016). Induction of broad-based immunity and protective efficacy by self-amplifying mRNA vaccines encoding influenza virus hemagglutinin. J. Virol..

[24] Heiser A (2002). Autologous dendritic cells transfected with prostate-specific antigen RNA stimulate CTL responses against metastatic prostate tumors. J. Clin. Investig..

[25] Wang Y (2013). Systemic delivery of modified mRNA encoding herpes simplex virus 1 thymidine kinase for targeted cancer gene therapy. Mol. Ther. J. Am. Soc. Gene Ther..

[26] Schirmacher V (2000). Intra-pinna anti-tumor vaccination with self-replicating infectious RNA or with DNA encoding a model tumor antigen and a cytokine. Gene Ther..

[27] Liu Z (2013). The multiple chemokine-binding bovine herpesvirus 1 glycoprotein G (BHV1gG) inhibits polymorphonuclear cell but not monocyte migration into inflammatory sites. Mol. Med..

[28] Mori E (2002). Essential role of monocyte chemoattractant protein-1 in development of restenotic changes (neointimal hyperplasia and constrictive remodeling) after balloon angioplasty in hypercholesterolemic rabbits. Circulation.

[29] Ni W (2001). New anti-monocyte chemoattractant protein-1 gene therapy attenuates atherosclerosis in apolipoprotein E-knockout mice. Circulation.

[30] Wada T (2004). Gene therapy via blockade of monocyte chemoattractant protein-1 for renal fibrosis. J Am. Soc. Nephrol..

[31] Spencer-Green G (2000). Etanercept (Enbrel): update on therapeutic use. Ann. Rheum. Dis..

[32] Mease PJ (2000). Etanercept in the treatment of psoriatic arthritis and psoriasis: a randomised trial. Lancet.

[33] Tekstra J (1996). Identification of the major chemokines that regulate cell influxes in peritoneal dialysis patients. J. Am. Soc. Nephrol..

[34] Topley N (1993). Human peritoneal mesothelial cells synthesize interleukin-8. Synergistic induction by interleukin-1 beta and tumor necrosis factor-alpha. Am. J. Pathol..

[35] Witowski J (2001). Synthesis of C-X-C and C-C chemokines by human peritoneal fibroblasts: induction by macrophage-derived cytokines. Am. J. Pathol..

[36] Matsukawa A (1999). Endogenous monocyte chemoattractant protein-1 (MCP-1) protects mice in a model of acute septic peritonitis: cross-talk between MCP-1 and leukotriene B4. J. Immunol..

[37] Montesinos MC, Desai A, Cronstein BN (2006). Suppression of inflammation by low-dose methotrexate is mediated by adenosine A2A receptor but not A3 receptor activation in thioglycollate-induced peritonitis. Arthr. Res. Ther..

[38] Immordino ML, Dosio F, Cattel L (2006). Stealth liposomes: review of the basic science, rationale, and clinical applications, existing and potential. Int. J. Nanomed..

[39] Proudfoot AE (2003). Glycosaminoglycan binding and oligomerization are essential for the in vivo activity of certain chemokines. Proc. Natl. Acad. Sci. USA.

[40] Koenen RR, Weber C (2010). Therapeutic targeting of chemokine interactions in atherosclerosis. Nat. Rev. Drug Discov..

[41] Severin IC (2012). Properties of 7ND-CCL2 are modulated upon fusion to Fc. Protein Eng. Des. Sel. PEDS.

[42] Harvey RD (2017). Science of biosimilars. J. Oncol. Pract..

[43] Hinz T (2017). The European regulatory environment of RNA-based vaccines. Methods Mol. Biol. (Clifton N.J.).

[44] Manchekar M, Liu Y, Sun Z, Richardson PE, Dashti N (2015). Phospholipid transfer protein plays a major role in the initiation of apolipoprotein B-containing lipoprotein assembly in mouse primary hepatocytes. J. Biol. Chem..

[45] Pertoft H, Johnsson A, Wärmegård B, Seljelid R (1980). Separation of human monocytes on density gradients of Percoll. J. Immunol. Methods.

