# Supplementary material for: Viromers as carriers for mRNA-mediated expression of therapeutic molecules under inflammatory conditions
Source: Sci Rep. 2020 Sep 15;10:15090. doi: 10.1038/s41598-020-72004-8 (PMC7494895; doi:10.1038/s41598-020-72004-8)
Supplement: Supplementary file 1 — Supplementary Information. [file 41598_2020_72004_MOESM1_ESM.pdf]

Supplementary information to

Viromers as carriers for mRNA-mediated expression of therapeutic biological molecules under inflammatory conditions

Edith Jansig, Stefanie Geissler, Vera Rieckmann, Anja Kuenemund, Benjamin Hietel, Mathias Schenk, Sebastian Wussow, Patrick Kreideweiss, Steffen Panzner, Christian Reinsch and Holger Cynis

This file contains:

- Supplementary Table 1
- Supplementary Fig. 1-9

Supplementary Table 1. Physicochemical characterization of Viromer/CCL2 and Viromer/FLuc complexes. Shown are the particle sizes and the polydispersity indices (PDI), both measured by dynamic light scattering (DLS), Zeta potential determined by laser Doppler electrophoresis (LDE) and accessible RNA as determined by Ribogreen assay.

**Parameter**	**Method**	**Viromer/CCL2 complexes**		**Viromer/Fluc complexes**	
		**ave.**	**SD**	**ave.**	**SD**
**Particle Size [nm]**	DLS	256	5	270	5
**PDI**	DLS	0.24	0.05	0.27	0.07
**Zeta potential [mV]**	LDE	-35.0	3.2	-11.9	0.1
**accessible RNA [%]**	Ribogreen	5.3	3.7	7.5	1.2

ave.: average; SD: Standard Deviation

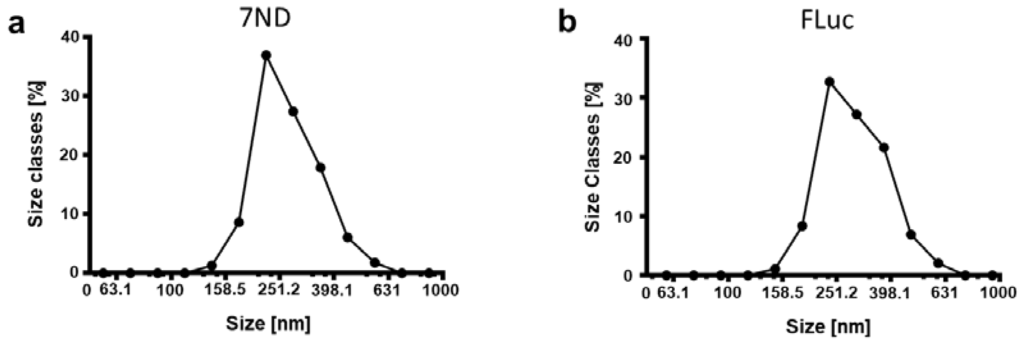

Supplementary Figure 1. Size classes of (**a**) Viromer/7ND and (**b**) Viromer/FLuc complexes as determined by dynamic light scattering.

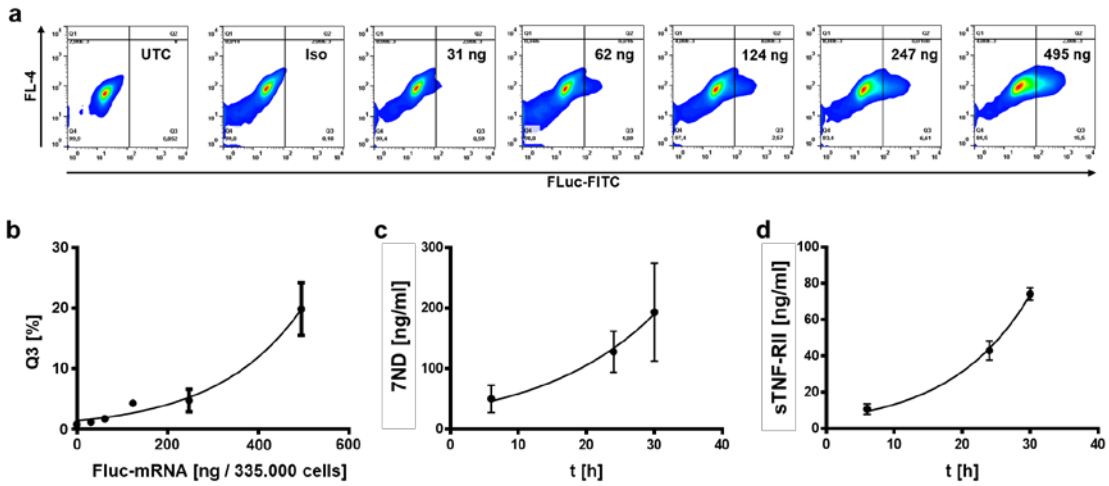

Supplementary Figure 2. Dose-dependent transfection of RAW264.7 macrophages using FLuc-mRNA. (**a**) FACS plots showing FLuc-positive cells in dependence of applied mRNA amount. For the experiment, 335.000 cells / well were seeded into a 24-well plate. (**b**) FLuc-positivity as function of applied mRNA amount. Highest efficiency was achieved by application of 495 ng FLuc-mRNA / 335.000 cells. Mean±SEM, n=2. (**c**) Exponential fit for the increase of 7ND in cell culture supernatant of RAW264.7, as presented in Fig. 1f. (**d**) Exponential fit for the increase of sTNF-RII in cell culture supernatant of RAW264.7, as presented in Fig. 1g.

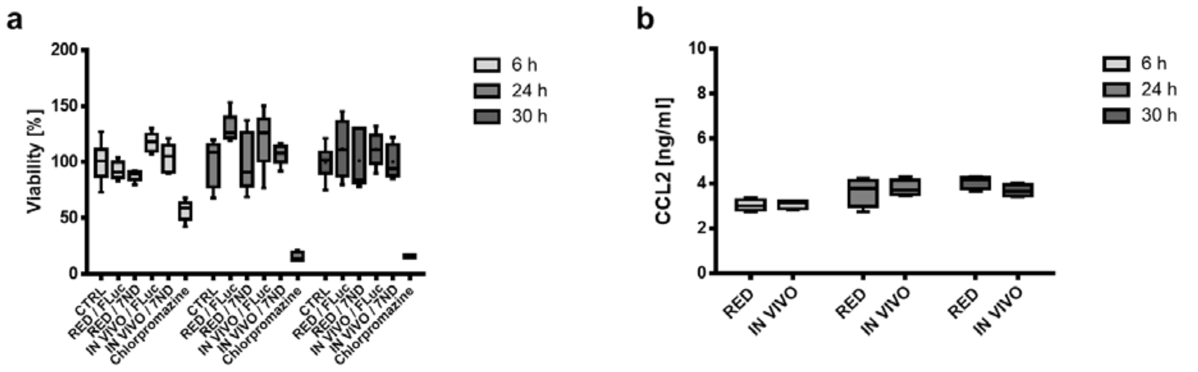

Supplementary Figure 3. Viability and CCL2 induction. (**a**) Viability of RAW264.7 cells was analysed after application of four different combinations of Viromer/mRNA complexes (RED/FLuc, RED/7ND, IN VIVO/FLuc, IN VIVO/7ND, (100 ng mRNA/well)). Viability was determined by measuring intracellular LDH using the Cyto-Tox-ONE homogenous membrane integrity assay (Promega) in comparison to control (Viromer buffer, CTRL) and Chlorpromazine (100 µM)-treated cells serving as low-viability control. (**b**) Analysis of CCL2 secreted from RAW264.7 cells after application of FLuc complexed with Viromer RED or Viromer IN VIVO. CCL2 generation was determined after 6 h, 24 h and 30 h. Viromer/FLuc complexes did not induce CCL2 secretion. (n=4).

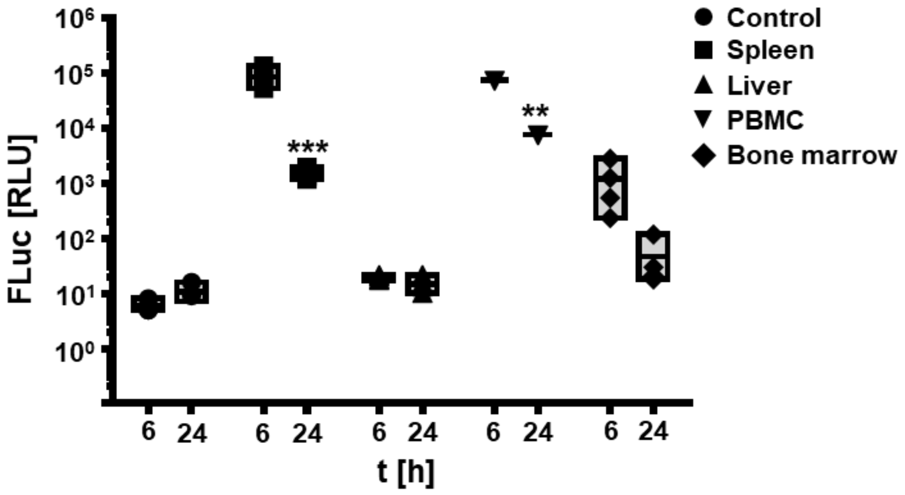

Supplementary Figure 4. Quantification of FLuc expression in different tissues after i.p. application of Viromer/FLuc-mRNA-complexes. Graphs displayed as floating bars (min to max). Line represents the mean. ** P<0.01, *** P<0.001, two-way ANOVA, Sidak multiple comparison test. n=1 (PBMC), n=4 (control spleen, liver, bone marrow).

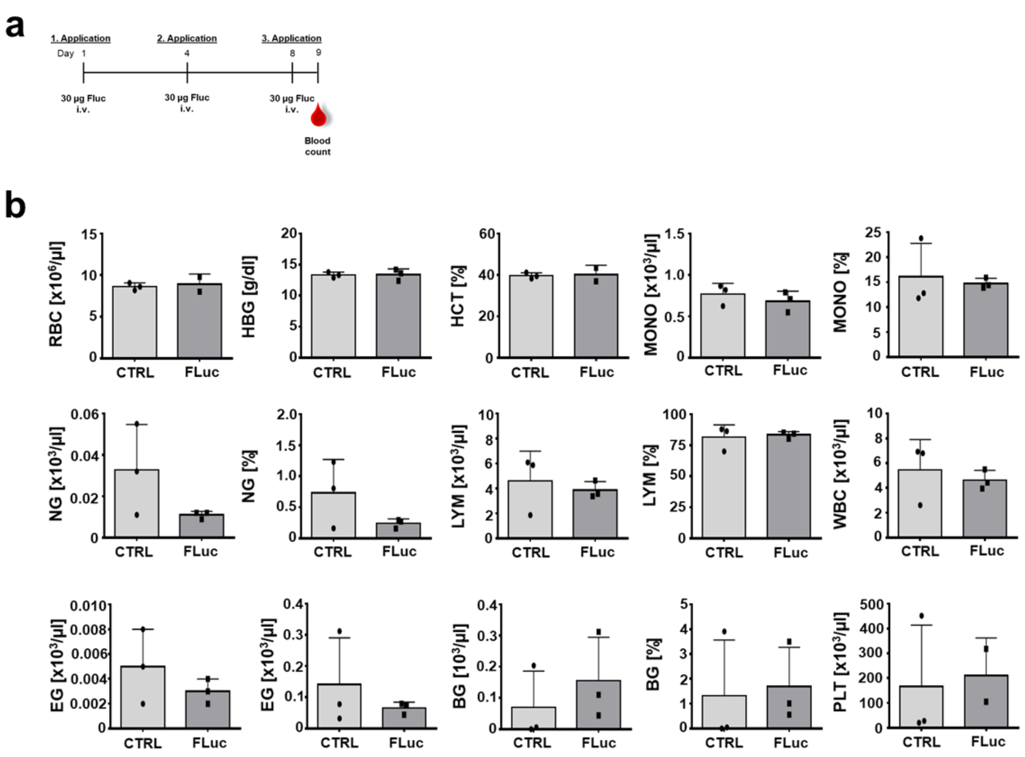

Supplementary Figure 5. Cellular blood count. (**a**) Timeline for the analysis of blood cells following 3 consecutive Viromer IN VIVO/FLuc applications (30 µg, i.v.) on days 1, 4 and 8 of the experiment. Blood count was determined 24 h after last i.v. dosing. (**b**) Differential blood count (n=2-3). RBC; red blood cells; HGB: haemoglobin; HCT: haematocrit; MONO: monocytes; NG: neutrophils; LYM: lymphocytes; WBC: white blood cells; EG: eosinophils; BG: basophils; PLT: platelets

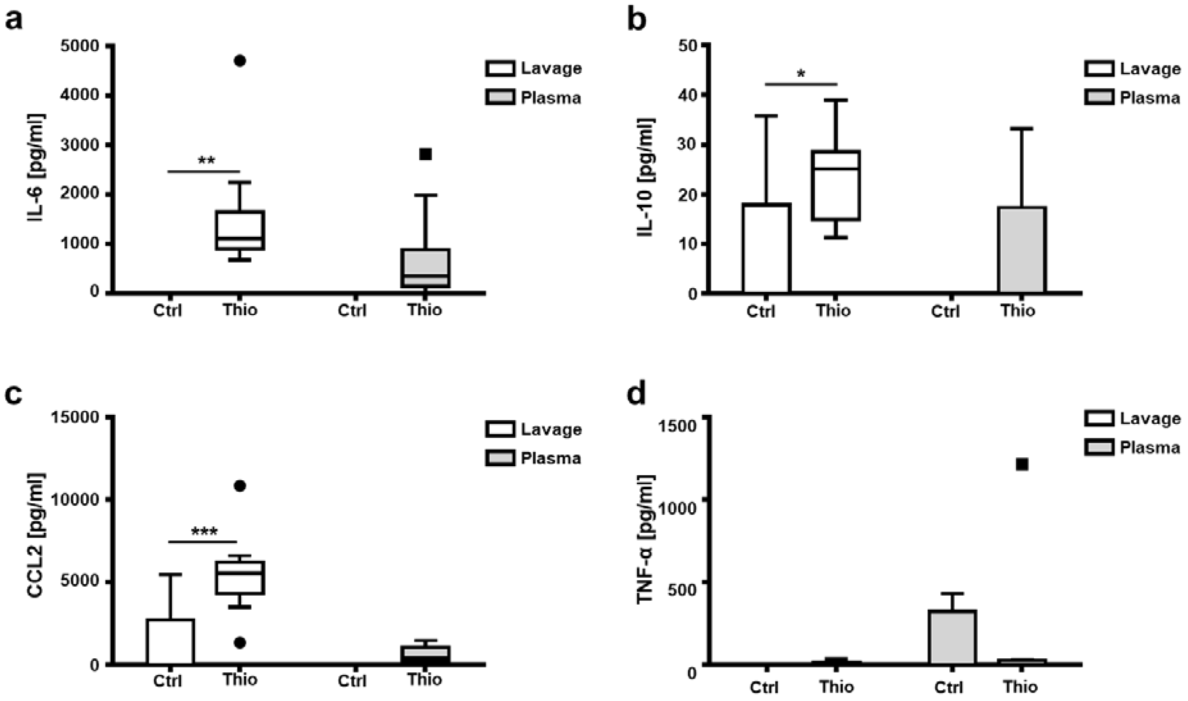

Supplementary Figure 6. Cytokine levels in lavage and plasma after thioglycolate challenge (Thio) in comparison to buffer control (Ctrl=PBS). (**a-d**) Analysis of cytokine levels of IL-6 (**a**), IL-10 (**b**), CCL2 (**c**) and TNF-alpha (**d**) using Bio-Plex. Box and whiskers (Tukey). Line represents mean. *P<0.05, **P<0.01, ***P<0.001, two-way ANOVA, Sidak multiple comparison test. n=4 (Ctrl), n=15 (Thio).

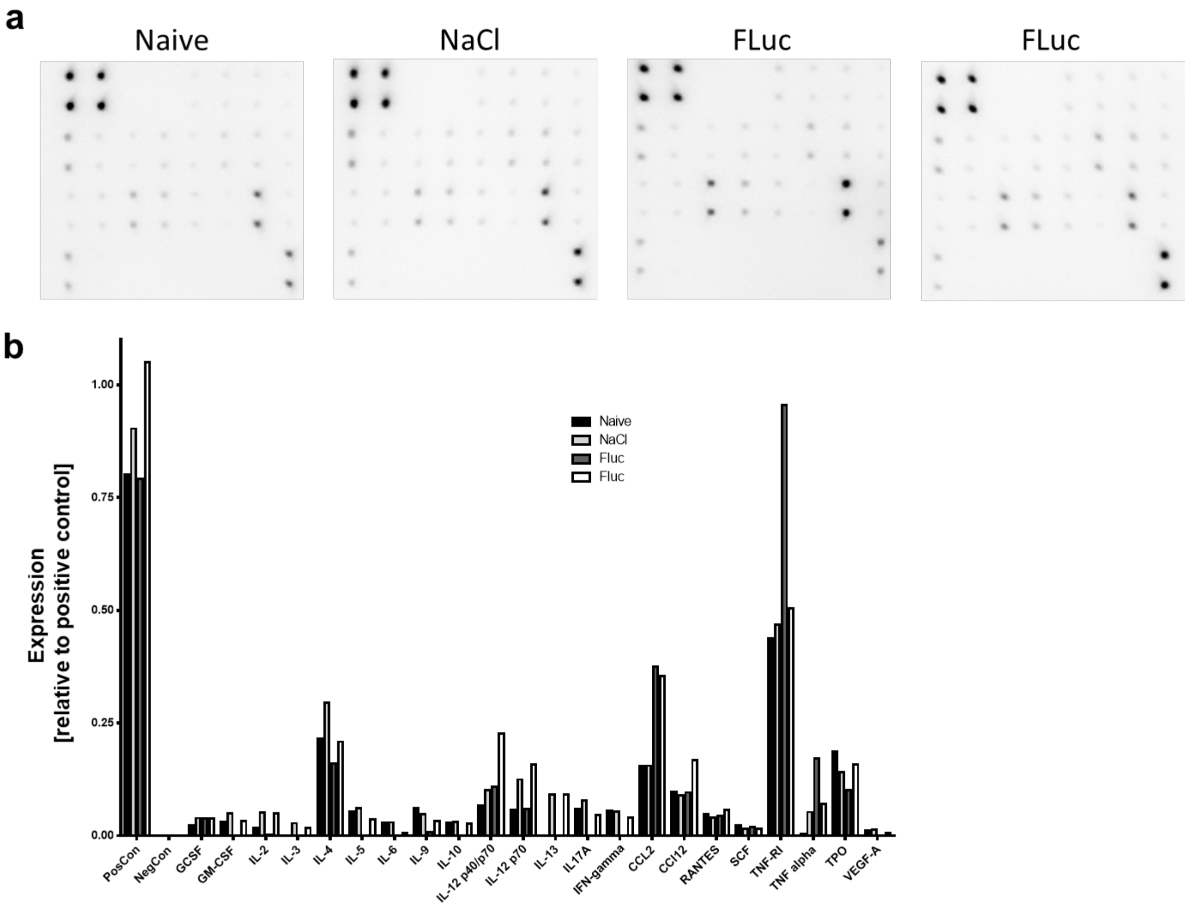

Supplementary Figure 7. Cytokine array for plasma cytokines after application of Viromer IN VIVO/FLuc complexes. (**a**) Cytokine arrays visualizing the cytokine profile of 22 different cytokines in plasma from 1 naïve mouse, 1 NaCl-injected mouse and 2 FLuc (30 µg/mouse)-injected mice. Injection scheme followed the scheme used for induction of thioglycolate-induced peritonitis with mice receiving NaCl or FLuc 6 hours prior to a second NaCl-injection. Analysis was 10 h after FLuc-injection. (**b**) Graphical illustration derived from (**a**). Dot intensity was quantified using ImageJ software. Blanks were subtracted from all samples. Depicted is grey scale level of each analyte relative to positive control.

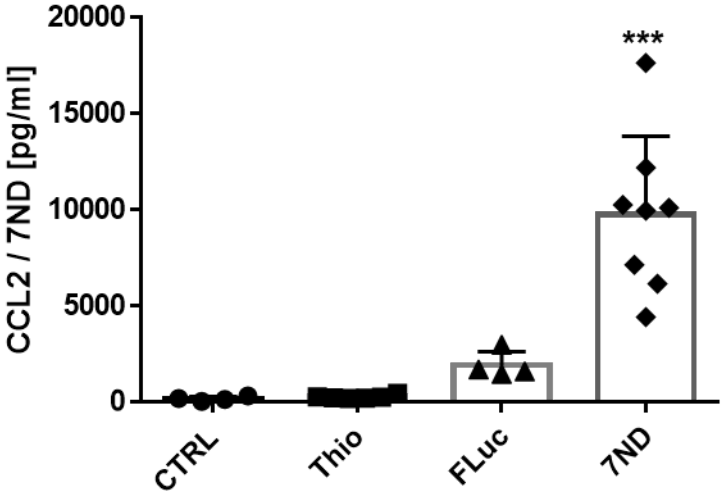

Supplementary Figure 8. Quantification of CCL2/7ND in plasma after application of thioglycolate (Thio), thioglycolate and 7ND (7ND), NaCl (CTRL) and FLuc. The figure is a collage of 2 independent experiments (CTRL / FLuc and Thio / 7ND). Thio and 7ND are also shown in Fig. 4g. Note the slight induction of CCL2 by FLuc application. Only 7ND leads to substantial amounts of CCL2/7ND in plasma. ****P*<0.001 vs. FLuc, one-way ANOVA, Sidak multiple comparison test. n=4 (CTRL, FLuc), n=6 (Thio), n=8 (7ND).

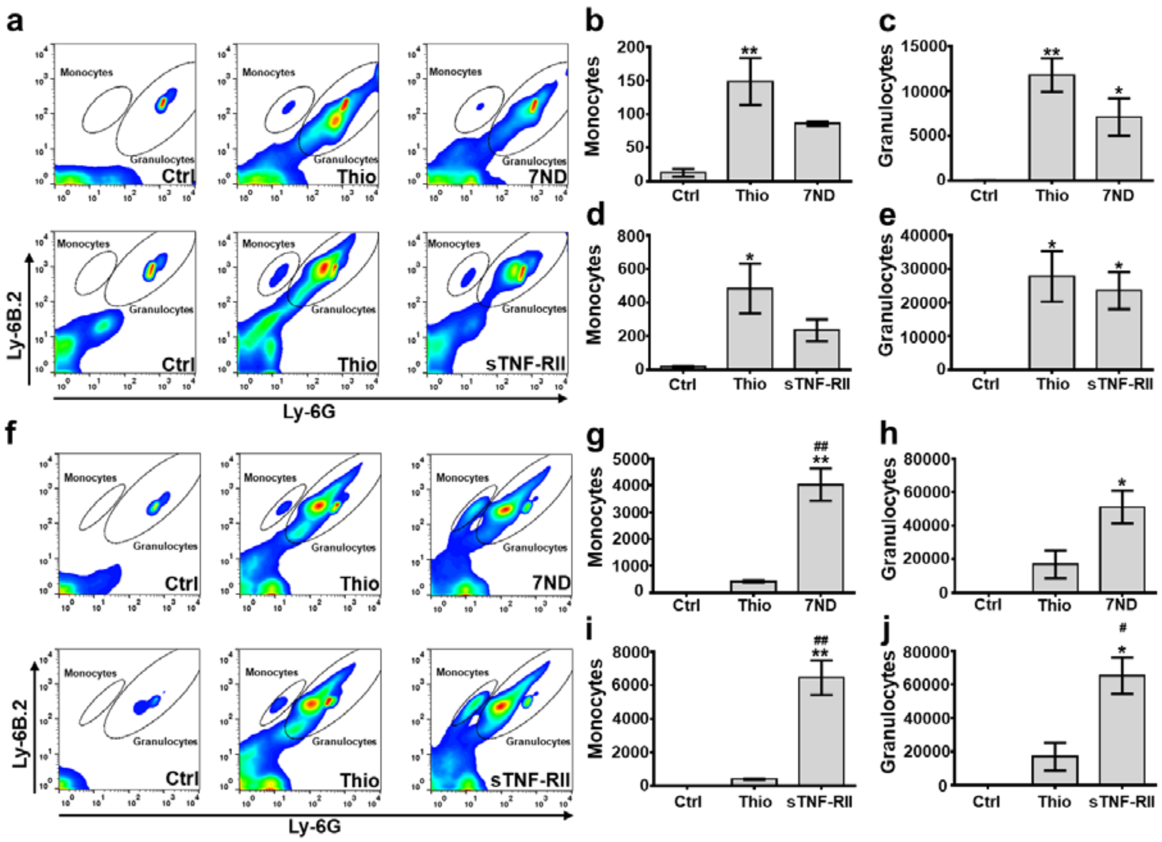

Supplementary Figure 9. Application of 7ND and sTNF-RII complexes depending on application route *in vivo*. (**a**) Representative FACS plots for the intravenous application of 7ND and sTNF-RII complexes. Gates for quantification of individual cell populations are marked in the plots. Positive events in Ctrl within the Granulocyte gate are the applied Trucount beads, which were subtracted from the total number of events for quantification. (**b-e**) Infiltrating monocytes (**b,d)** and granulocytes (**c,e)** were quantified for both, 7ND (**b,c**) and sTNF-RII (**d,e**) application. Mean±SEM. **P*<0.05, ***P*<0.01 vs. ctrl, one-way ANOVA, Tukey multiple comparison test, n=3-4. (**f**) Representative FACS plots for the intraperitoneal application of 7ND and sTNF-RII complexes. **(g-j**) Infiltrating monocytes (**g,i)** and granulocytes (**h,j)** were quantified for both, 7ND (**g,h**) and sTNF-RII (**i,j**) application. Mean±SEM. * *P*<0.05, ***P*<0.01 vs. ctrl, ## *P*<0.01 vs. Thio, one-way ANOVA, Tukey multiple comparison test, n=2-3.
